# Effective nutrition support for patients with chronic obstructive pulmonary disease: managing malnutrition in primary care

**DOI:** 10.3399/bjgp21X717053

**Published:** 2021-08-27

**Authors:** Peter F Collins, Anita Nathan, Shelley Roberts, Tom Wilkinson

**Affiliations:** Department of Dietetics & Food Services, Mater Health, Brisbane, QLD, Australia; Mater Research Institute, University of Queensland, South Brisbane, QLD, Australia.; GPs with Interest in Nutrition and Lifestyle Group (GPING) RCGP Specialist Group; GP representative, ‘Managing Adult Malnutrition in the Community’ and ‘Managing Malnutrition in COPD’ consensus panels, UK.; School of Health Sciences and Social Work, Griffith University, Southport, QLD, Australia; Menzies Health Institute Queensland, Griffith University, Southport, QLD, Australia; Allied Health Research, Gold Coast Hospital and Health Service, Southport, QLD, Australia.; Clinical and Experimental Sciences, Faculty of Medicine, University of Southampton, NIHR Southampton Biomedical Research Centre, UK.

The prevalence of malnutrition is high among patients living with chronic obstructive pulmonary disease (COPD), affecting around 1 in 3 inpatients and 1 in 5 outpatients.^1^ Its consequences are profound, including poorer quality of life,^1^ increased emergency healthcare use, prolonged episodes of hospitalisation, early readmission, and increased health and social care costs.^1^

## IS WEIGHT LOSS AN INEVITABLE CONSEQUENCE OF THE PROGRESSIVE NATURE OF COPD?

**Figure 1.**
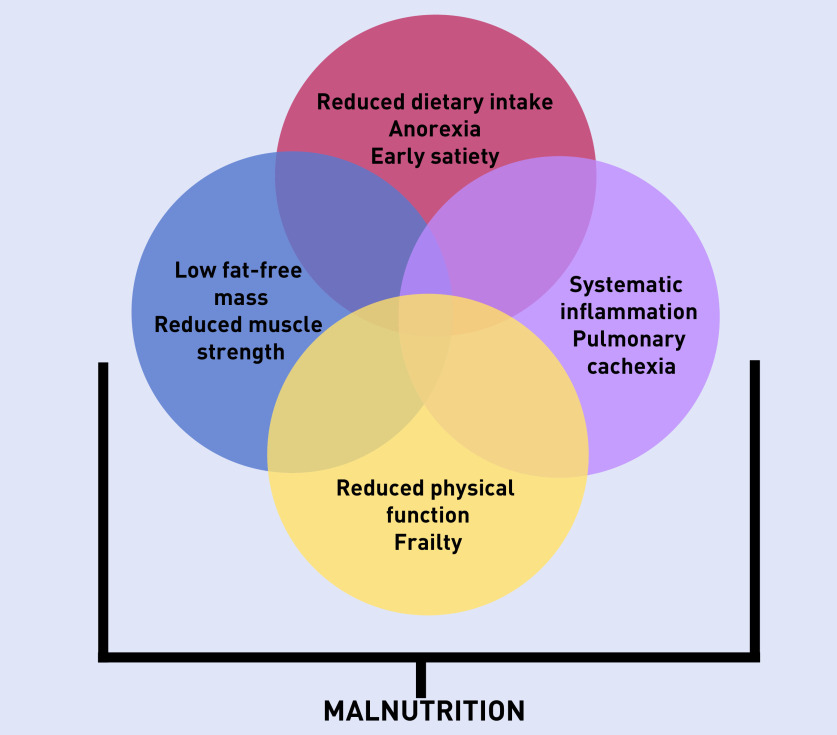
***Complex aetiology of malnutrition in COPD. Source: adapted from Collins et al 2019.****3* ***COPD = chronic obstructive pulmonary disease.***

The aetiology of malnutrition in COPD is complex, multifactorial (Figure 1), and is likely to be bidirectional, with malnutrition being both a cause and a consequence of severe respiratory disease. Research has also shown that malnutrition is significantly and independently associated with social deprivation regardless of COPD disease severity.^2^

While patients with COPD can present with varying predominance of the respiratory phenotypes, that is, chronic bronchitis and emphysema, all patients may experience functional symptoms such as reduced exercise tolerance, and nutrition impact symptoms including anorexia, shortness of breath, and early satiety. A poor nutritional intake is therefore likely commonplace in patients with COPD, particularly during exacerbations of the disease.^3^

The evolution in our understanding of the systemic nature of COPD (that is, related to systematic inflammation and underlying pathophysiology) has resulted in a number of nutritional phenotypes being identified, which can be characterised by physical and metabolic abnormalities;^4^ these can present both in isolation or combination, including malnutrition, sarcopenia, and frailty. A key disabling feature is the progressive decline in exercise capacity due to reduced size and function of skeletal muscle. This change in muscle mass and function is often not clinically apparent, as overall body mass can be maintained with excess fat mass masking clinically relevant muscle loss.

When energy intake is insufficient to meet daily needs, weight loss arises, resulting in depletion of fat stores and reduced muscle synthesis. During exacerbations, reduced nutritional intake is often accompanied by increased inflammation, pharmacotherapy such as corticosteroids, and reduced physical activity.^3^ These factors combine to further contribute to a negative nitrogen balance, reduced protein synthesis, and increased muscle breakdown.

## HOW SHOULD WE BE IDENTIFYING AND MANAGING MALNUTRITION IN PRIMARY CARE?

Routine screening using a validated tool (for example, the Malnutrition Universal Screening Tool [MUST]) and surveillance are key National Institute for Health and Care Excellence (NICE) recommendations^5^ and should be embedded into all health and social care settings. Body mass index (BMI) should be calculated for all patients with COPD, with particular attention paid to unintentional changes in weight exceeding 5% in the previous 3 months or greater than 10% over 6 months.^1^

Weight loss could indicate significant other pathology, but should be managed at the same time as further investigations into the loss of weight. For those at risk of malnutrition, further assessment should be undertaken to identify contributory causes. Nutrition support should include dietary advice (including food fortification), and advice on assistance with eating and/or texture-modified diets where appropriate. The use of oral nutritional supplements (ONS) is recommended by NICE for those with BMI <20 kg/m^2^ or for rapid weight loss.^1^^,^^5^

## WHAT IS THE ROLE OF NUTRITION SUPPORT AND ONS IN EFFECTIVE TREATMENT OF COPD?

Nutrition support should be an integral part of COPD management across the continuum of care.^4^^,^^5^ Systematic reviews and meta-analyses investigating the effectiveness of nutrition support in COPD have found that malnutrition is amenable to nutrition intervention, which leads to significant improvements in nutritional intake and nutritional status,^6^ as well as improved functional capacity, respiratory muscle strength, and quality of life.^7^ The majority of evidence for nutrition support in the treatment of COPD-related malnutrition is based on ready-to-drink (RTD) ONS in stable outpatients,^6^^,^^7^ and is currently lacking for other forms of nutrition support such as powdered ONS and enteral tube feeding.

Based on this evidence, several important considerations should be highlighted:

- if the goal is to treat malnutrition, low-volume RTD ONS for up to 12 weeks, in combination with dietary advice, should be commenced and monitored^1^ in consultation with a dietitian where available;
- as patients respond to nutrition support and their intake and nutritional status improves, they should be transitioned to a powdered ONS and a high-energy, high-protein diet;
- powdered ONS in combination with individualised dietary counselling can be effective at treating malnutrition in stable (non-exacerbating) patients with COPD;^8^ however, as consumption appears to be considerably lower than RTD ONS,^6^ patients should be reviewed regularly to monitor adherence and response to treatment;
- where patients are not responding to nutrition intervention strategies (for example, poor compliance and/or worsening malnutrition), referral to a dietitian is recommended;^1^ and
- among those who are malnourished, a 2 kg increase is suggested as a threshold at which functional improvements are seen.^7^ Timelines to achieve weight gain will depend on the individual’s condition and a resolution of malnutrition can take months rather than weeks.

## SUMMARY

GPs can help to identify patients with COPD who are at risk of malnutrition (for example, during routine reviews and when managing acute exacerbations). Screening and subsequent identification of at-risk patients should prompt early intervention when patients are more stable and more likely to respond to any nutrition support provided.

Although the evidence for nutrition support in managing malnutrition in COPD is almost entirely based on RTD ONS, due to increased pressure on NHS prescribing budgets, the use of RTD ONS is often delayed in preference for a ‘food first’ approach and/ or powdered ONS. The authors believe that, for patients at high risk of malnutrition, an assertive, evidence-based, step-down nutrition support approach is required, as these patients need early intervention for the best outcomes.

The evidence and recommendations presented in this article have been incorporated into the updated Managing Malnutrition in COPD pathway (www.malnutritionpathway.co.uk/copd),^1^ which aims to assist professionals in identifying and managing malnutrition. Patient leaflets are also available, according to nutritional status, risk, and disease status. It is hoped this article and the pathway will facilitate opportunities to integrate dietitians into primary care who can support decision making and provide training where needed. This should lead to quality improvement in nutrition care in community settings, through the identification of malnutrition, the start of earlier patient conversations about diet and nutrition, and confident initiation of appropriate nutrition support where clinically indicated.

## References

[1] Anderson L, Banner J, Bostock B (2020). Managing malnutrition in COPD.

[2] Collins PF, Elia M, Kurukulaaratchy RJ, Stratton RJ (2018). The influence of deprivation on malnutrition risk in outpatients with chronic obstructive pulmonary disease (COPD). Clin Nutr.

[3] Collins PF, Yang IA, Chang Y-C, Vaughan A (2019). Nutritional support in chronic obstructive pulmonary disease (COPD): an evidence update. J Thorac Dis.

[4] Schols AM, Ferreira IM, Franssen FM (2014). Nutritional assessment and therapy in COPD: a European Respiratory Society statement. Eur Respir J.

[5] National Institute for Health and Care Excellence (2018). Chronic obstructive pulmonary disease in over 16s: diagnosis and management NG115.

[6] Collins PF, Stratton RJ, Elia M (2012). Nutritional support in chronic obstructive pulmonary disease: a systematic review and meta-analysis. Am J Clin Nutr.

[7] Collins PF, Hukins C, Allport C (2018). Nutritional support in chronic obstructive pulmonary disease (COPD): a randomised trial. Clin Nutr.

[8] Collins PF, Elia M, Stratton RJ (2013). Nutritional support and functional capacity in chronic obstructive pulmonary disease: a systematic review and meta-analysis. Respirology.

